# Ambient fine particulate matter and allergic symptoms in the middle-aged and elderly population: results from the PIFCOPD study

**DOI:** 10.1186/s12931-023-02433-2

**Published:** 2023-05-25

**Authors:** Shanshan Wei, Jiping Liao, Tao Xue, Kunyao Yu, Xiuhua Fu, Ruiying Wang, Xiaomin Dang, Cheng Zhang, Hua Qiao, Shujuan Jiang, Jianhong Xiao, Lixia Dong, Jinzhi Yin, Xixin Yan, Weihua Jia, Guifang Zhang, Rui Chen, Bo Zhou, Beibei Song, Jing Li, Mengyu Yin, Lina Zhang, Liping Xie, Shaochen Dong, Jian Sun, Peng Gao, Bifang Miao, Wei Li, Lan He, Qian Ning, Limin Zhao, Hengyi Liu, Han Cao, Guangfa Wang

**Affiliations:** 1 Department of Respiratory and Critical Care Medicine, Peking University First Hospital, No.8 Xishiku Street, Xicheng District, Beijing, 100034 China; 2 Institute of Reproductive and Child Health/National Health Commission Key Laboratory of Reproductive Health and Department of Epidemiology and Biostatistics, School of Public Health, Peking University Health Science Centre, Beijing, China; 3 Division of Pulmonary and Critical Care Medicine, The Affiliated Hospital of Inner Mongolia Medical University, Hohhot, Inner Mongolia China; 4 Department of Pulmonary and Critical Care Medicine, Shanxi Bethune Hospital, Shanxi Academy of Medical Sciences, Tongji Shanxi Hospital, Third Hospital of Shanxi Medical University, Taiyuan, Shanxi China; 5 Respiratory and Critical Care Medicine, Xi’an Jiaotong University Medical College First Affiliated Hospital, Xi’an, Shaanxi China; 6 The First Hospital of Qinhuangdao, Qinhuangdao, Hebei China; 7 Department of Respiratory and Critical Care Medicine, Shandong Provincial Hospital Affiliated to Shandong First Medical University, Jinan, Shandong China; 8 Mindong Hospital of Ningde City, Ningde, Fujian China; 9 Tianjin Medical University General Hospital, Tianjin, China; 10 The Second Hospital of Jilin University, Changchun, Jilin China; 11 Department of Respiratory and Critical Care Medicine, The Second Hospital of Hebei Medical University, Shijiazhuang, Hebei China; 12 Hebei Key Laboratory of Respiratory Critical Care, Shijiazhuang, Hebei China; 13 General Hospital of Taiyuan Iron & Steel (Group) Co., LTD, Taiyuan, Shanxi China; 14 Jinyuan Community Health Service Center, Taiyuan, Shanxi China; 15 Jining First People’s Hospital, Jining, Shandong China; 16 Henan Provincial People’s Hospital, Zhengzhou, Henan China; 17 Department of Biostatistics, Peking University First Hospital, Beijing, China

**Keywords:** Ambient fine particulate matter, Allergic symptoms, Short- and long-term exposure

## Abstract

**Background:**

The associations between short- and long-term exposure to ambient fine particulate matter with an aerodynamic diameter ≤ 2.5 µm (PM_2.5_) and allergic symptoms in middle-aged and elderly populations remain unclear, particularly in China, where most cities have severe air pollution.

**Methods:**

Participants (n = 10,142; age = 40–75 years) were recruited from ten regions in China from 2018 to 2021 for the Predictive Value of Inflammatory Biomarkers and Forced Expiratory Volume in 1 s (FEV_1_) for Chronic Obstructive Pulmonary Disease (PIFCOPD) study. Short-term (lag0 and lag0–7 day) and long-term (1-, 3- and 5-year) PM_2.5_ concentrations at residences were extracted from the air pollutant database known as Tracking Air Pollution (TAP) in China. Multivariate logistic regression models were used to estimate associations for short- and long-term PM_2.5_ exposure concentrations and long-term exposure models were additionally adjusted for short-term deviations.

**Results:**

A 10 µg/m^3^ increase in PM_2.5_ on the day the allergic symptoms questionnaire was administered (lag0 day) was associated with higher odds of allergic nasal (1.09, 95% CI 1.05, 1.12) and eye symptoms (1.08, 95% CI 1.05, 1.11), worsening dyspnea caused by allergens (1.06, 95% CI 1.02, 1.10), and ≥ 2 allergic symptoms (1.07, 95% CI 1.03, 1.11), which was similar in the lag0–7 day concentrations. A 10 µg/m^3^ increase in the 1-year average PM_2.5_ concentration was associated with an increase of 23% for allergic nasal symptoms, 22% for eye symptoms, 20% for worsening dyspnea caused by allergens, and 21% for ≥ 2 allergic symptoms, similar to the 3- and 5-year average PM_2.5_ concentrations. These associations between long-term PM_2.5_ concentration and allergic symptoms were generally unchanged after adjustment for short-term deviations.

**Conclusions:**

Short- and long-term exposure to ambient PM_2.5_ was associated with an increased risk of allergic nasal and eye symptoms, worsening dyspnea caused by allergens, and ≥ 2 allergic symptoms.

***Trial registration*:**

Clinical trial ID: NCT03532893 (29 Mar 2018).

**Supplementary Information:**

The online version contains supplementary material available at 10.1186/s12931-023-02433-2.

## Background

Ambient fine particulate matter with an aerodynamic diameter ≤ 2.5 µm (PM_2.5_) is detrimental to public health [1, 2]. Over the past decades, there has been growing evidence that ambient PM_2.5_ exposure is a risk factor for developing and exacerbating asthma and allergic diseases [1, 3–8]. The global population-weighted PM_2.5_ concentration increased from 39.7 µg/m^3^ in 1990 to 44.2 µg/m^3^ in 2015 [2]. There is growing concern that ambient PM_2.5_ may contribute to the prevalence of allergic diseases and symptoms.

With rapid economic development, urbanization, and industrialization in recent decades, China has become one of the most polluted countries worldwide [2]. PM_2.5_ concentrations increased from 1990 and peaked during 2011–2013 [9]. In the wake of the air pollution crisis, the State Council of China promulgated the toughest-ever Air Pollution Prevention and Control Action Plan (APPCAP) in 2013 [10]. China initiated reductions in anthropogenic PM_2.5_ emissions in 2013, and population-weighted PM_2.5_ concentrations rapidly decreased by 4.51 µg/m^3^/year from 2013 to 2016 [11]. However, as of the end of 2017, the entire Chinese population lived in areas with annual average PM_2.5_ concentrations exceeding 10 µg/m^3^ [12] (World Health Organization (WHO) interim target 4), and 81.1% lived in areas with concentrations above 35 µg/m^3^ [13] (Chinese grade I ambient air-quality standard) [9]. In contrast to other countries, China is currently transitioning from high to low air pollution levels, which provides an excellent opportunity to study the effects of air pollution on human health.

Short-term exposure to air pollution can exacerbate preexisting asthma [1], allergic diseases [4], and chronic obstructive pulmonary disease (COPD) [14] and cause an increased risk of hospitalization or even mortality related to respiratory and allergic diseases. Long-term exposure to air pollution increases the risk of morbidity and mortality from asthma [1, 8] and allergic diseases [6, 15]. However, some studies did not report evidence of a positive association between ambient air pollution and asthma and allergic diseases [16, 17]. There is an urgent need to investigate the impact of short- and long-term air pollutant exposure on allergic symptoms in a large well-documented cohort with detailed covariate data.

Most published studies on ambient PM_2.5_ exposure, allergic symptoms, and asthma were mainly performed in regions with relatively low air pollution concentrations [15, 18, 19] and young populations, such as children, adolescents, and young adults [16, 17]. However, more than 90% of air pollution-related deaths occur in Asia and Africa, where air pollution is generally a serious problem [10, 20]. In recent decades, the prevalence of allergic diseases such as asthma and allergic rhinoconjunctivitis has been increasing worldwide among older adults [8, 21]. The national cross-sectional China Pulmonary Health (CPH) study revealed that the prevalence of asthma increased with age, from 2.5% in individuals aged 20 to 39 years to 5.4% in those aged 40 years or older [21]. China, one of the countries most burdened by air pollution, is home to a fifth of the world’s older people [22]. However, there is a lack of data with respect to the effect of ambient PM_2.5_ exposure on allergic symptoms among middle-aged and elderly populations. Considering the burden of allergic diseases attributed to ambient air pollution exposure, a better understanding of whether middle-aged and elderly individuals are susceptible to this exposure should enable the design of prevention strategies.

Therefore, this study aimed to estimate the associations between allergic symptoms and short- and long-term ambient PM_2.5_ exposure in the Predictive Value of Inflammatory Biomarkers and Forced Expiratory Volume in 1 s (FEV_1_) for COPD (PIFCOPD) study, a nationwide prospective cohort study in China.

## Methods

### Study design and participants

The PIFCOPD study, an ongoing nationwide prospective cohort study, enrolled 10,385 middle-aged and elderly individuals without respiratory disease in China between May 2018 and October 2021. The studied population covered ten regions: Beijing, Jilin, Inner Mongolia, Shandong, Fujian, Hebei, Shanxi, Tianjin, Shaanxi, and Henan. Details of the study have been previously described [23]. After excluding individuals with ages outside the 40–75 year range (n = 113) and those with at least one missing covariate (n = 130), 10,142 participants (3756 men and 6386 women) were included in this study.

The study was approved by the ethics review committees of the Peking University First Hospital (2018 Number 31). Written informed consent was acquired from all participants.

### Procedures

Data were collected at local branch health centers. Participants underwent medical examinations, including anthropometric measurements, spirometry, blood tests, and urinary tests. A series of standard self-administered questionnaires, including demographic, risk factors, lifestyle questionnaires, St George’s Respiratory Questionnaire (SGRQ), and the allergic symptoms questionnaire [24] were administered by trained reviewers. In this study, data were collected on the nonperiod-specified prevalence of allergic nasal (“Do you sometimes have an itchy, runny, or stuffy nose?”) and eye symptoms (“Do you sometimes have itchy, watery, swollen or burning eyes?”), wheezing (“Do you ever experience wheezing?”) and worsening dyspnea caused by allergens (“Does exposure to dust, pollen, or pets make your shortness of breath worse?”). Education level was classified as no schooling or primary school, middle school, high school, and college or higher. Body mass index (BMI) was calculated by taking the participant’s weight in kilograms divided by their height in meters squared. Passive smoking was defined as the exposure of a nonsmoker to smoke at home or in a work setting. Biomass exposure was defined as the daily use of wood or animal waste fuels for cooking or heating. Occupational exposure was defined as working in environments with remarkably harmful dust or fumes.

### Exposure assessment

Ambient PM_2.5_ concentrations, extracted from the air pollutant database known as Tracking Air Pollution in China (TAP) [25], were estimated based on a two-stage machine learning model coupled with the synthetic minority oversampling technique and a tree-based gap-filling method. Daily PM_2.5_ concentrations in China with complete coverage at a spatial resolution of 10 km have been available since 2000. Details of the PM_2.5_ exposure assessment model have been described in our previous publications [11]. This model is comparable to models in other studies, and the average out-of-bag cross-validation R^2^ for different years was 0.83 [26]. Residential PM_2.5_ exposure concentrations were estimated by assigning predicted PM_2.5_ concentrations to each participant’s residential address. We defined short-term exposure as the daily average of a single 0 day (lag0 day) and the moving average of 0-to-7 day concentrations (lag0–7 day) before the day before the allergic symptoms questionnaire was administered. Long-term PM_2.5_ exposure was estimated by calculating the 1-, 3- and 5-year averages of the PM_2.5_ concentrations for the calendar year before administering the allergic symptoms questionnaire. We also obtained the daily average of the ambient temperature on the day the allergic symptoms questionnaire was administered (lag0 day) at a 9 km × 9 km spatial resolution from the atmospheric reanalysis V5 (ERA5) Land dataset.

### Statistical analyses

We compared baseline characteristics between the < 2 allergic symptoms group and the ≥ 2 allergic symptoms group using the independent sample test-test for continuous variables and the χ^2^ test for categorical variables. Multivariable logistic regression models were used to estimate associations between short- (lag0 and lag0–7 day) and long-term (1-, 3- and 5-year) PM_2.5_ concentrations at residences and self-reported allergic nasal and eye symptoms, worsening dyspnea caused by allergens and ≥ 2 allergic symptoms. Effect estimates are presented as odds ratios (ORs) and their 95% confidence intervals (CIs) for each 10 µg/m^3^ increase in the PM_2.5_ concentration. We gradually added potential confounders to observe their effects, and a total of four models were developed: Model 1, no adjustments; Model 2, adjusted for age, sex, education level (no schooling or primary school, middle school, high school, and college or higher), BMI (< 25 kg/m^2^, 25–29.9 kg/m^2^, ≥ 30 kg/m^2^), geographic region (north, east, northeast, northwest), ambient temperature (lag0 day, average on the day the allergic symptoms questionnaire was administered), the season in which the allergic symptoms questionnaire was administered (winter and summer, spring and autumn); Model 3, further adjusted for cumulative smoking exposure (0, 1–19, ≥ 20 pack-years), passive smoking, biomass exposure, household cooking, and occupational exposure; and Model 4 (only for long-term exposure models), further adjusted for short-term PM_2.5_ concentration deviations. In Model 4, we examined the influence of additional adjustments for short-term variations in long-term concentrations when estimating associations between allergic symptoms and long-term PM_2.5_ exposure, including the 1-, 3- and 5-year average concentrations. Short-term PM_2.5_ concentration deviations were defined for each participant as the absolute difference between the PM_2.5_ concentration on the day the allergic symptoms questionnaire was administered and the 1-, 3- and 5-year average concentrations. For example, the short-term PM_2.5_ concentration deviation for the model of the 1-year average PM_2.5_ concentration was calculated as follows: Short-term PM_2.5_ concentration deviation = PM_2.5_ concentration on the day the allergic symptoms questionnaire was administered—1-year average PM_2.5_ concentration. The associations between ambient PM_2.5_ exposure and wheezing were not analyzed in this study because the prevalence of wheezing was low, and the regression model could not be run after adjusting for a range of covariates.

All statistical analyses were performed using R version 4.1.2. All two-tailed tests were regarded as statistically significant at the 0.05 level.

## Results

The distribution of 10,142 participants (6386 females and 3756 males) by general characteristics and risk factors is summarized in Table 1. The participants had a mean age of 59.27 years (range 40–75 years), most (65%) lived in the northern region, more than half (58%) cooked at home, and only 4.9% had biomass exposure. Among the participants, 9721 had one or no allergic symptoms, and the other 421 (4.2%) had more than one allergic symptom. There were significant differences in education level, passive smoking, smoking type, cumulative smoking exposure (pack-years), household cooking, occupational exposure, family history of asthma, geographic region, and season between the two groups. The proportions of passive smokers (18% vs. 9.3%) and former or current smokers (24% vs. 19%) were higher in the ≥ 2 allergic symptoms group than in the < 2 allergic symptoms group. For education level, the proportion of participants with a high education level was low in the < 2 allergic symptoms group. A total of 256 (2.6%) of the 9721 participants in the < 2 allergic symptoms group and 39 (9.3%) of the 421 participants in the ≥ 2 allergic symptoms group had a family history of asthma. The proportion of participants who completed the allergic symptoms questionnaire in autumn was also higher in the ≥ 2 allergic symptoms group than in the < 2 allergic symptoms group (28% vs. 18%). There was a higher proportion of household cooking (71% vs. 58%) and occupational exposure (17% vs. 7.2%) in the ≥ 2 allergic symptoms group. For allergic symptoms, eye symptoms had the highest prevalence (6.1%), followed by nasal symptoms (5.6%), worsening dyspnea caused by allergens (3.2%), and wheezing (1.2%) (Table 2).

**Table 1:** Demographics and risk factors by allergic symptoms in the PIFCOPD population (N = 10,142)

	ALL	< 2 allergic symptoms	≥ 2 allergic symptoms	p-value
	N = 10,142	N = 9721	N = 421	
Sex				0.797
Female	6386 (63)	6118 (63)	268 (64)	
Male	3756 (37)	3603 (37)	153 (36)	
Age, y	59.27 (8.41)	59.28 (8.42)	59.03 (8.17)	0.549
BMI group				0.684
< 25 kg/m^2^	5938 (59)	5691 (59)	247 (59)	
25–29.9 kg/m^2^	3548 (35)	3405 (35)	143 (34)	
≥ 30 kg/m^2^	656 (6.5)	625 (6.4)	31 (7.4)	
Education level				< 0.001
No schooling or primary school	1824 (18)	1795 (18)	29 (6.9)	
Middle school	4555 (45)	4409 (45)	146 (35)	
High school	2387 (24)	2236 (23)	151 (36)	
College or higher	1376 (14)	1281 (13)	95 (23)	
Passive smoking	975 (9.6)	901 (9.3)	74 (18)	< 0.001
Smoking type				0.003
Never smoker	8234 (81)	7916 (81)	318 (76)	
Former or current smoker	1908 (19)	1805 (19)	103 (24)	
Cumulative smoking exposure, pack-years				0.011
0	8234 (81)	7916 (81)	318 (76)	
1–19	706 (7.0)	668 (6.9)	38 (9.0)	
≥ 20	1202 (12)	1137 (12)	65 (15)	
Biomass exposure	495 (4.9)	466 (4.8)	29 (6.9)	0.063
Household cooking	5896 (58)	5599 (58)	297 (71)	< 0.001
Occupational exposure	771 (7.6)	699 (7.2)	72 (17)	< 0.001
Family history of asthma	295 (2.9)	256 (2.6)	39 (9.3)	< 0.001
Geographic region				< 0.001
North	6627 (65)	6283 (65)	344 (82)	
East	1678 (17)	1647 (17)	31 (7.4)	
Northeast	768 (7.6)	724 (7.4)	44 (10)	
Northwest	1069 (11)	1067 (11)	2 (0.5)	
Season^a^				< 0.001
Winter	373 (3.7)	369 (3.8)	4 (1.0)	
Spring	1594 (16)	1525 (16)	69 (16)	
Summer	6325 (62)	6095 (63)	230 (55)	
Autumn	1850 (18)	1732 (18)	118 (28)	

Data are presented mean (SD) for continuous variables and n (%) for categorical variables

^a^Season in which the allergic symptoms questionnaire was administered. BMI, body mass index

**Table 2:** Allergic symptoms prevalence in the PIFCOPD study population

Questionnaire items	N (%)
Allergic nasal symptoms	
Does not sometimes have an itchy, runny, or stuffy nose	9576 (94)
Does sometimes have an itchy, runny or stuffy nose	566 (5.6)
Allergic eye symptoms	
Does not sometimes have itchy, watery, swollen or burning eyes	9520 (94)
Does sometimes have itchy, watery, swollen or burning eyes	622 (6.1)
Wheezing	
Has not had wheezing symptoms	10,022 (99)
Has had wheezing symptoms	120 (1.2)
Worsening dyspnea caused by allergens	
Has not had worsened shortness of breath due to exposure to dust, pollen, or pets	9813 (97)
Has had worsened shortness of breath due to exposure to dust, pollen or pets	329 (3.2)
≥ 2 allergic symptoms	421 (4.2)

Data are presented as n (%)

The average residential PM_2.5_ concentration was 35.19 (27.32) µg/m^3^ (range 1–288 µg/m^3^) on the day the allergic symptoms questionnaire was administered (lag0 day) and 34.73 (20.93) µg/m^3^ (range 2.00–197.12 µg/m^3^) for the moving average of 0-to-7 day (lag0–7 day). For long-term exposure, the 1-, 3- and 5-year annual average PM_2.5_ concentrations were 50.56 (15.27) µg/m^3^, 54.08 (16.92) µg/m^3^, and 59.79 (17.88) µg/m^3^, respectively (Table 3). The results of our study showed that during 2013–2020, the annual average concentrations of PM_2.5_ among ten regions were 81.97 µg/m^3^, 73.55 µg/m^3^, 67.21 µg/m^3^, 62.35 µg/m^3^, 58.53 µg/m^3^, 49 µg/m^3^, 46.09 µg/m^3^, and 44.92 µg/m^3^, respectively. The annual average PM_2.5_ concentration decreased by 45.2% from 2013 (81.97 µg/m^3^) to 2020 (44.92 µg/m^3^) among the ten regions (Additional file 1: Fig. S1). PM_2.5_ concentrations exhibited seasonal variation, with the highest concentration in winter (90.08 µg/m^3^, 5-year average), followed by autumn (56.26 µg/m^3^), spring (55.33 µg/m^3^), and summer (44.99 µg/m^3^) (Additional file 1: Fig. S2).

**Table 3:** Distribution of the estimated ambient temperature and PM_2.5_ concentrations at residence

	Mean (SD)	Median (IQR)	Range
Short-term PM_2.5_, µg/m^3^			
Lag0 day	35.19 (27.32)	30.00 (25.00)	1.00 to 288.00
Lag0–7 day	34.73 (20.93)	31.75 (22.00)	2.00 to 197.12
Long-term PM_2.5_, µg/m^3^			
1-year	50.56 (15.27)	49.84 (20.47)	10.93 to 86.50
3-year	54.08 (16.92)	53.09 (23.64)	13.43 to 93.32
5-year	59.79 (17.88)	59.41 (23.71)	15.45 to 100.35
Ambient temperature^a^, ℃	20.81 (7.88)	23.08 (7.61)	− 19.03 to 32.76

^a^Average on the day the allergic symptoms questionnaire was administered. PM_2.5_, particulate matter with an aerodynamic diameter ≤ 2.5 µm

The results from the three models (Model 1, Model 2, and Model 3) all showed that allergic symptoms increased with higher short-term PM_2.5_ exposure (Table 4). In adjusted Model 3, the ORs of allergic symptoms per 10 µg/m^3^ increase in lag0 day average PM_2.5_ concentration were 1.09 (95% CI 1.05, 1.12) for allergic nasal symptoms, 1.08 (95% CI 1.05, 1.11) for allergic eye symptoms, 1.06 (95% CI 1.02, 1.10) for worsening dyspnea caused by allergens, and 1.07 (95% CI 1.03, 1.11) for ≥ 2 allergic symptoms. The effect estimates were similar to those obtained using the PM_2.5_ data of the lag0–7 day concentrations.

**Table 4:** Associations between short-term PM_2.5_ exposures and allergic symptoms in the PIFCOPD study population

	Mode 1		Model 2		Model 3	
	OR (95% CI)	p-value	OR (95% CI)	p-value	OR (95% CI)	p-value
Allergic nasal symptoms						
Lag0 day	1.08 (1.06, 1.11)	< 0.001	1.09 (1.06, 1.12)	< 0.001	1.09 (1.05, 1.12)	< 0.001
Lag0–7 day	1.1 (1.06, 1.14)	< 0.001	1.11 (1.06, 1.16)	< 0.001	1.11 (1.06, 1.16)	< 0.001
Allergic eye symptoms						
Lag0 day	1.08 (1.05, 1.10)	< 0.001	1.08 (1.05, 1.11)	< 0.001	1.08 (1.05, 1.11)	< 0.001
Lag0–7 day	1.13 (1.09, 1.16)	< 0.001	1.15 (1.11, 1.20)	< 0.001	1.16 (1.11, 1.20)	< 0.001
Worsening dyspnea caused by allergens						
Lag0 day	1.07 (1.04, 1.10)	< 0.001	1.06 (1.02, 1.10)	0.002	1.06 (1.02, 1.10)	0.002
Lag0–7 day	1.09 (1.04, 1.13)	< 0.001	1.06 (1.00, 1.11)	0.049	1.06 (1.00, 1.12)	0.06
≥ 2 allergic symptoms						
Lag0 day	1.07 (1.04, 1.10)	< 0.001	1.07 (1.04, 1.10)	< 0.001	1.07 (1.03, 1.11)	< 0.001
Lag0–7 day	1.09 (1.05, 1.13)	< 0.001	1.09 (1.03, 1.14)	0.001	1.09 (1.03, 1.14)	0.002

Effect estimates are presented as odds ratios (ORs) and their 95% confidence intervals (CIs) for each 10 µg/m^3^ increase in the PM_2.5_ concentration. Model 1, no adjustments; Model 2, adjusted for age, sex, education level (no schooling or primary school, middle school, high school, and college or higher), BMI (< 25 kg/m^2^, 25–29.9 kg/m^2^, ≥ 30 kg/m^2^), geographic region (north, east, northeast, northwest), ambient temperature (lag0 day, average on the day the allergic symptoms questionnaire was administered), the season in which the allergic symptoms questionnaire was administered (winter and summer, spring and autumn); Model 3, further adjusted for cumulative smoking exposure (0, 1–19, ≥ 20 pack-years), passive smoking, biomass exposure, household cooking, and occupational exposure. PM_2.5_, particulate matter with an aerodynamic diameter ≤ 2.5 µm

There were positive associations between long-term PM_2.5_ concentrations (1-, 3- and 5-year average) and allergic symptoms (Table 5). Compared with the 3- and 5-year average PM_2.5_ concentrations, the 1-year average PM_2.5_ concentration had a stronger impact on allergic symptoms in the four long-term exposure models. In Model 3, each 10 µg/m^3^ increase in the 1-year average PM_2.5_ concentration was associated with higher odds of allergic nasal (1.23, 95% CI 1.14, 1.33) and eye symptoms (1.22, 95% CI 1.13, 1.31), worsening dyspnea caused by allergens (1.20, 95% CI 1.09, 1.32) and ≥ 2 allergic symptoms (1.21, 95% CI 1.11, 1.32); these effect estimates were similar to those after adjustment for short-term PM_2.5_ concentration deviations in Model 4. Specifically, the OR of allergic eye symptoms for a 10 µg/m^3^ increase in the 1-year average concentration was 1.22 (95% CI 1.13, 1.31) based on Model 3, compared with that of 1.22 (95% CI 1.14, 1.32) in Model 4 after adjustment for short-term PM_2.5_ concentration deviations. Compared with long-term PM_2.5_ concentration, the short-term PM_2.5_ concentration deviations had a weaker impact on allergic symptoms in model 4. Each 10 µg/m^3^ increase in the 1-year short-term PM_2.5_ concentration deviation, the risk increased by 7% for allergic nasal symptoms (1.07, 95% CI 1.04, 1.10), 6% for allergic eye symptoms (1.06, 95% CI 1.03, 1.09), 5% for worsening dyspnea caused by allergens (1.05, 95% CI 1.01, 1.09), 6% for ≥ 2 allergic symptoms (1.06, 95% CI 1.02, 1.10), these effect estimates were similar to those in 3- and 5-year average PM_2.5_ concentration logistic regression models (Additional file 1: Tables S3–S5).

**Table 5:** Associations between long-term PM_2.5_ exposures and allergic symptoms in the PIFCOPD study population

	Model 1		Model 2		Model 3		Model 4	
	OR (95% CI)	p-value	OR (95% CI)	p-value	OR (95% CI)	p-value	OR (95% CI)	p-value
Allergic nasal symptoms								
1-Year	1.25 (1.18, 1.33)	< 0.001	1.25 (1.17, 1.35)	< 0.001	1.23 (1.14, 1.33)	< 0.001	1.23 (1.14, 1.33)	< 0.001
3-Year	1.14 (1.09, 1.20)	< 0.001	1.14 (1.07, 1.22)	< 0.001	1.13 (1.06, 1.21)	< 0.001	1.14 (1.07, 1.22)	< 0.001
5-Year	1.14 (1.09, 1.20)	< 0.001	1.12 (1.05, 1.19)	< 0.001	1.11 (1.04, 1.19)	0.001	1.13 (1.06, 1.20)	< 0.001
Allergic eye symptoms								
1-Year	1.23 (1.16, 1.30)	< 0.001	1.24 (1.16, 1.33)	< 0.001	1.22 (1.13, 1.31)	< 0.001	1.22 (1.14, 1.32)	< 0.001
3-Year	1.17 (1.11, 1.23)	< 0.001	1.2 (1.12, 1.27)	< 0.001	1.2 (1.12, 1.28)	< 0.001	1.21 (1.13, 1.29)	< 0.001
5-Year	1.17 (1.12, 1.23)	< 0.001	1.17 (1.11, 1.24)	< 0.001	1.17 (1.10, 1.25)	< 0.001	1.19 (1.12, 1.26)	< 0.001
Worsening dyspnea caused by allergens								
1-Year	1.26 (1.17, 1.37)	< 0.001	1.2 (1.10, 1.32)	< 0.001	1.2 (1.09, 1.32)	< 0.001	1.2 (1.09, 1.32)	< 0.001
3-Year	1.17 (1.09, 1.25)	< 0.001	1.09 (1.01, 1.19)	0.033	1.1 (1.01, 1.20)	0.032	1.1 (1.01, 1.20)	0.026
5-Year	1.17 (1.10, 1.25)	< 0.001	1.08 (1.00, 1.17)	0.057	1.08 (1.00, 1.17)	0.062	1.09 (1.00, 1.18)	0.041
≥ 2 allergic symptoms								
1-Year	1.24 (1.16, 1.33)	< 0.001	1.22 (1.12, 1.33)	< 0.001	1.21 (1.11, 1.32)	< 0.001	1.21 (1.11, 1.32)	< 0.001
3-Year	1.14 (1.08, 1.21)	< 0.001	1.11 (1.03, 1.20)	0.006	1.11 (1.03, 1.21)	0.006	1.12 (1.04, 1.21)	0.004
5-Year	1.15 (1.08, 1.21)	< 0.001	1.09 (1.02, 1.17)	0.012	1.1 (1.02, 1.18)	0.015	1.11 (1.03, 1.19)	0.007

Effect estimates are presented as odds ratios (ORs) and their 95% confidence intervals (CIs) for each 10 µg/m^3^ increase in the PM_2.5_ concentration. Model 1, no adjustments; Model 2, adjusted for age, sex, education level (no schooling or primary school, middle school, high school, and college or higher), BMI (< 25 kg/m^2^, 25–29.9 kg/m^2^, ≥ 30 kg/m^2^), geographic region (north, east, northeast, northwest), ambient temperature (lag0 day, average on the day the allergic symptoms questionnaire was administered), the season in which the allergic symptoms questionnaire was administered (winter and summer, spring and autumn); Model 3, further adjusted for cumulative smoking exposure (0, 1–19, ≥ 20 pack-years), passive smoking, biomass exposure, household cooking, and occupational exposure; and Model 4, further adjusted for short-term PM_2.5_ concentration deviations. PM_2.5_, particulate matter with an aerodynamic diameter ≤ 2.5 µm

In addition to PM_2.5_ exposure, the ORs for the other covariates were similar in short- and long-term PM_2.5_ concentrations models (Additional file 1: Tables S1–S5). Specifically, multivariable logistic regression showed that ≥ 2 allergic symptoms was associated with older age (1.03, 95% CI 1.01, 1.04), higher education level (middle school (2.20, 95% CI 1.46, 3.41); high school (3.38, 95% CI 2.24, 5.27); college or higher (4.34, 95% CI 2.78, 6.96)), passive smoking (2.22, 95% CI 1.65, 2.97), household cooking (1.53, 95% CI 1.20, 1.96), occupational exposure (1.42, 95% CI 1.06, 1.88) and family history of asthma (2.95, 95% CI 2.02, 4.21) in the 1-year average PM_2.5_ concentration logistic regression model 4 (Additional file 1: Table S3), which was similar to that of allergic nasal and eye symptoms, and worsening dyspnea caused by allergens.

## Discussion

In the PIFCOPD study, short- and long-term ambient PM_2.5_ exposure levels were significantly associated with allergic symptoms, apart from other individual risk factors, including older age, higher education level, passive smoking, household cooking, occupational exposure, and family history of asthma. The associations between PM_2.5_ exposure and allergic symptoms, including allergic nasal and eye symptoms, worsening dyspnea caused by allergens, and ≥ 2 allergic symptoms, were stronger for the 1-, 3- and 5-year concentrations than the lag0 day, and lag0–7 day concentrations. Adjusting for short-term PM_2.5_ concentration deviations had little effect on the estimated associations with long-term exposures. Our study provides new evidence of the health effects of ambient PM_2.5_ exposure in relation to the prevalence of allergic symptoms in the middle-aged and elderly Chinese population.

China is experiencing a transition period from high to low air pollution levels. The PM_2.5_ concentration trends in our study were consistent with previous reports [27, 28]. As the focus of the APPCAP, a remarkable decline in the annual average PM_2.5_ concentration was achieved [28], with yearly concentrations decreasing by 45.2% (from 2013 to 2020) in our study. However, the short- and long-term exposure concentrations were considerably higher than the current Chinese grade I ambient air-quality standard [13] or the WHO interim target 4 [12] in this study. Winter is the most severe season for ambient PM_2.5_ air pollution [27]. A total of 8464 (83.5%) participants lived in northern, northeastern, and northwestern geographic regions, where coal burning is needed for heating in winter, emitting massive anthropogenic air pollutants.

Consistent with our study, previous studies showed that the prevalence of allergic symptoms and diseases increased when ambient air pollutants such as PM_2.5_ were high [21, 29, 30]. Experimental exposure to PM_2.5_ could directly impair the barrier function of epithelial cells and induce oxidative stress and inflammatory responses [31], thereby leading to allergic symptoms. Moreover, particulate matter contains many potential allergen carriers, such as pollutants, aerosols, pollens, bacteria, and fungi [32, 33], and interacts with allergens to enhance their immunogenicity [34, 35]. In addition, particulate matter also has an adjuvant effect on the production of immunoglobulin E (IgE) to common environmental allergens [36]. The synergistic biological effects induced by allergens and ambient PM_2.5_ could be one of the potential mechanisms illustrating the increasing risk of allergic diseases and symptoms at high PM_2.5_ exposure levels.

Air pollution contributes to the onset and aggravation of allergic symptoms or diseases [21, 35, 37]. Most previous studies were conducted on children and young adults [7, 19, 30]. However, few studies have examined the associations between ambient PM_2.5_ exposure and allergic nasal or eye diseases among middle-aged and elderly populations in large well-documented cohorts. Our study revealed that short- and long-term ambient PM_2.5_ exposure is positively associated with allergic nasal and eye symptoms among middle-aged and elderly populations. Consistent with our results, one study from Fukuoka showed that an interquartile range (IQR) increase in PM_2.5_ at lag0 day was associated with allergic nasal (1.08, 95% CI 1.03, 1.13) and eye (1.10, 95% CI 1.04, 1.16) symptoms among 2317 schoolchildren [7]. Ambient PM_2.5_ exposure might alter tear film stability, causing the film to break up and thin by disrupting the tear lipid layer, thus causing eye irritation and discomfort [38]. According to a hospital-based population study in Japan, the ambient PM_2.5_ concentration was associated with the number of outpatient visits for allergic conjunctivitis during the nonpollen season [4]. The China, Children, Homes and Health (CCHH) project revealed that each 10 µg/m^3^ increase in the annual PM_2.5_ concentration was associated with a 20% increase in the prevalence of allergic rhinitis among preschool children in six Chinese cities [30]. Short-term PM_2.5_ exposure levels were correlated with nasal lavage fluid eosinophils and exudation mediators in children with asthma in Paris [19]. PM_2.5_ can increase inflammatory cytokines, induce pathological damage to the nasal mucosa and conjunctival epithelium, and worsen nasal and eye symptoms [39, 40]. Improved air quality might reduce the inflammatory response and reduce allergic diseases and symptoms [41].

Exposure to high levels of ambient PM_2.5_ is a potential aggravating factor for allergic respiratory symptoms or diseases [1, 42]. Wheezing and worsening dyspnea caused by allergens are common allergic respiratory symptoms [43, 44]. The prevalence of allergic respiratory symptoms was 9–20.4% in a previous study [42, 44, 45], compared to that of 1.2% for wheezing and 3.2% for worsening dyspnea caused by allergens in this study. The discrepancy might be attributed to the exclusion of individuals with chronic respiratory diseases such as COPD, emphysema, asthma, and other conditions from this study. Consistent with previous studies [5, 42, 46], our study found a positive association between PM_2.5_ exposure and worsening dyspnea caused by allergens. A combined analysis of cross-sectional data from Lifelines and UK Biobank cohorts, each 5 µg/m^3^ increase in the PM_2.5_ concentration was associated with an increase of 16% for wheezing and 61% for shortness of breath [42]. PM_2.5_ exposure, either alone or in combination with allergic sensitization, can induce oxidative stress, signal transduction interference, enzyme inhibition, epigenetic dysregulation, airway hyperresponsiveness and remodeling [47]. Allergic respiratory symptoms can decrease with air quality improvement [48] and smoking cessation [49].

Our study found long-term PM_2.5_ exposure was more strongly associated with allergic symptoms than short-term PM_2.5_ exposure. The findings in our study could be attributable in part to differences between long- and short-term ambient PM2.5 exposure levels. Ambient PM_2.5_ exhibited the highest concentration in winter (90.08 µg/m^3^, 5-year average) and the lowest concentration in summer (44.99 µg/m^3^, 5-year average). Among the participants, 6325 (62%) were enrolled in the study in the summer, and only 373 (3.7%) were enrolled in the winter. The short-term (lag0 and lag0–7 day concentrations) ambient PM_2.5_ exposure levels were lower than the long-term (1-, 3- and 5-year average concentrations) in this study. Meanwhile, the questionnaire collected nonperiod-specified rather than recent allergic symptoms, which may be another important reason for the stronger associations for long-term than short-term PM_2.5_ exposure.

Short-term ambient PM_2.5_ concentration deviations might not significantly affect on long-term PM_2.5_ exposure models. Consistent with our results, a study of the LuftiBus cohort adjusting for short-term variations in nitrogen dioxide (NO_2_) and PM_2.5_ concentrations had little effect on the estimated associations between air pollution exposure and lung function parameters in long-term exposure models [50]. Other studies adjusted for previous single-day or moving average concentrations instead of short-term deviations and revealed that the conclusions of associations between lung function parameters and long-term air pollution exposure were not altered [51, 52]. Therefore, it might not be necessary to adjust for short-term air pollution concentrations, including short-term deviations, previous single-day or moving average concentrations, when estimating the effect of long-term ambient air pollution exposure.

Allergic diseases, which involve complex interactions of genetic, ethnic, environmental, and socioeconomic status or lifestyle risk factors, are primarily attributed to environmental factors such as indoor and outdoor air pollution, tobacco smoke exposure, and exposure to other pollutants [21, 37, 44, 53]. In addition to ambient PM_2.5_ exposure, we also found that allergic symptoms were positively associated with older age, higher education level, passive smoking, household cooking, occupational exposure and family history of asthma. Household cooking and tobacco smoke are major sources of indoor air pollution. Chinese cooking emits more PM_2.5_ than Western cooking [54]. For risk factors for allergic symptoms, previous studies have mainly focused on cooking fuel but not cooking itself [55]. In this study, a total of 58% (5896/10142) of the participants cooked frequently at home, only 4.9% (495/10142) had biomass exposure, and 84% (4969/5896) had kitchen ventilation. Household cooking itself, not cooking fuel, is a major risk factor for allergic symptoms. Despite smoking bans in public places in China, 9.6% of the participants in our study were still exposed to environmental tobacco smoke at home or in the workplace. We found that passive smoking was associated with increased allergic symptoms. Consistent with our results, a study based on the Respiratory Health in Northern Europe (RHINE) cohort revealed that passive smoking increased the risk of wheezing (1.26, 95% CI 1.02, 1.57) [56]. Family history of asthma is a well-known risk factor for asthma [53]. This study found that a history of asthma in close relatives is also a risk factor for nasal symptoms, eye symptoms, worsening dyspnea caused by allergens, and ≥ 2 allergic symptoms.

Several limitations of this study should be addressed. First, given the study design, it is challenging to provide causal inferences about the associations between PM_2.5_ exposure and allergic symptoms. Further intervention and prospective studies are needed to verify the causality of the association in this study. Second, allergic symptoms were assessed by self-report questionnaires, making the study prone to recall bias. Third, as an issue commonly reflected in other studies, PM_2.5_ exposure concentrations were only estimated at the residence due to a need for more information about work addresses or time-activity patterns. This might result in misclassification. Limited by data availability, information about other ambient pollutants was not available, and we could not distinguish between associations due to PM_2.5_ specifically or other correlated pollutants.

## Conclusions

In conclusion, the findings from the PIFCOPD study showed that short- and long-term ambient PM_2.5_ exposure might have adverse effects on allergic symptoms among the middle-aged and elderly population in China, apart from other individual risk factors, including older age, higher education level, passive smoking, household cooking, occupational exposure and family history of asthma. Our findings contribute substantially to the evidence of adverse effects of ambient PM_2.5_ exposure on allergic symptoms in middle-aged and elderly populations. This study further supports for the urgent need to control air pollution to protect middle-aged and elderly adults.

## Supplementary Information

**Additional file 1: Figure S1.** Trends in annual average PM2.5 concentrations among ten regions from 2013 to 2020 year. **Figure S2.** Season average PM2.5 concentrations of 1-, 3- and 5-year. **Table S1.** Estimated risks of each independent variable in the lag0 day PM2.5 concentration logistic regression model 3. **Table S2.** Estimated risks of each independent variable in the lag0-7 day PM2.5 concentration logistic regression model 3. **Table S3.** Estimated risks of each independent variable in the 1-year average PM2.5 concentration logistic regression model 4. **Table S4.** Estimated risks of each independent variable in the 3-year average PM2.5 concentration logistic regression model 4. **Table S5.** Estimated risks of each independent variable in the 5-year average PM2.5 concentration logistic regression model 4.

## Data Availability

Requests for data should be directed to the corresponding author. Patient-level data will be made available within 5 years of publication. Requests will be assessed before being granted. Data will be anonymized and securely transferred. A data-sharing agreement might be required.

## Abbreviations

APPCAP: Air Pollution Prevention and Control Action Plan
BMI: Body mass index
CIs: Confidence intervals
COPD: Chronic obstructive pulmonary disease
CPH: China Pulmonary Health
ERA5: The atmospheric reanalysis V5
FEV_1_: Forced expiratory volume in 1 s
IgE: Immunoglobulin E
IQR: Interquartile range
ORs: Odds ratios
PIFCOPD: Predictive Value of Inflammatory Biomarkers and FEV_1_ for COPD
PM_2.5_: Particulate matter with an aerodynamic diameter ≤ 2.5 µm
SGRQ: St George’s Respiratory Questionnaire
TAP: Tacking Air Pollution
WHO: World Health Organization
