# Supplementary material for: Ambient fine particulate matter and allergic symptoms in the middle-aged and elderly population: results from the PIFCOPD study
Source: Respir Res. 2023 May 25;24:139. doi: 10.1186/s12931-023-02433-2 (PMC10214547; doi:10.1186/s12931-023-02433-2)
Supplement: Supplementary file 1 — Additional file 1: Figure S1. Trends in annual average PM2.5 concentrations among ten regions from 2013 to 2020 year. Figure S2. Season average PM2.5 concentrations of 1-, 3- and 5-year. Table S1. Estimated risks of each independent variable in the lag0 day PM2.5 concentration logistic regression model 3. Table S2. Estimated risks of each independent variable in the lag0-7 day PM2.5 concentration logistic regression model 3. Table S3. Estimated risks of each independent variable in the 1-year average PM2.5 concentration logistic regression model 4. Table S4. Estimated risks of each independent variable in the 3-year average PM2.5 concentration logistic regression model 4. Table S5. Estimated risks of each independent variable in the 5-year average PM2.5 concentration logistic regression model 4. [file 12931_2023_2433_MOESM1_ESM.docx]

Supplement materials

Figure S1: Trends in annual average PM_2.5_ concentrations among ten regions from 2013 to 2020 year.

PM_2.5_, particulate matter with an aerodynamic diameter of ≤ 2.5 µm.

Figure S2: Season average PM_2.5_ concentrations of 1-, 3- and 5-year.

PM_2.5_, particulate matter with an aerodynamic diameter of ≤ 2.5 µm.

**Table S1: Estimated risks of each independent variable in the lag0 day PM_2.5_ concentration logistic regression model 3.**

|  | Allergic nasal symptoms | | Allergic eye symptoms | | Worsening dyspnea caused  by allergens | | ≥ 2 allergic symptoms | |
| --- | --- | --- | --- | --- | --- | --- | --- | --- |
|  | OR (95%CI) | p-value | OR (95%CI) | p-value | OR (95%CI) | p-value | OR (95%CI) | p-value |
| Lag0 day PM_2.5_, per 10 µg/m^3^ | 1.09(1.05, 1.12) | <0.001 | 1.08(1.05, 1.11) | <0.001 | 1.06(1.02, 1.10) | 0.002 | 1.07(1.03, 1.11) | <0.001 |
| Male | 1.29(1.00, 1.65) | 0.046 | 1.11(0.87, 1.41) | 0.4 | 0.83(0.59, 1.15) | 0.3 | 1.02(0.75, 1.36) | >0.9 |
| Age, y | 1.03(1.01, 1.04) | <0.001 | 1.01(1.00, 1.02) | 0.023 | 1.02(1.01, 1.04) | 0.002 | 1.03(1.01, 1.04) | <0.001 |
| BMI group |  |  |  |  |  |  |  |  |
| < 25 kg/m^2^ | 1 |  | 1 |  | 1 |  | 1 |  |
| 25-29.9 kg/m2 | 0.83(0.68, 1.00) | 0.047 | 0.91(0.76, 1.08) | 0.3 | 1.16(0.92, 1.47) | 0.2 | 0.9(0.72, 1.12) | 0.3 |
| ≥ 30 kg/m2 | 0.92(0.63, 1.30) | 0.6 | 1.2(0.87, 1.63) | 0.3 | 1.23(0.78, 1.87) | 0.3 | 1.08(0.72, 1.58) | 0.7 |
| Education level |  |  |  |  |  |  |  |  |
| Nonschooling or primary school | 1 |  | 1 |  | 1 |  | 1 |  |
| Middle school | 1.57(1.13, 2.22) | 0.009 | 1.81(1.33, 2.49) | <0.001 | 1.29(0.86, 1.98) | 0.2 | 2.16(1.44, 3.35) | <0.001 |
| High school | 2.46(1.76, 3.50) | <0.001 | 2.31(1.68, 3.23) | <0.001 | 2.05(1.36, 3.18) | <0.001 | 3.48(2.31, 5.43) | <0.001 |
| College or higher | 3.48(2.42, 5.08) | <0.001 | 3.19(2.26, 4.57) | <0.001 | 3.15(2.01, 5.02) | <0.001 | 4.45(2.85, 7.13) | <0.001 |
| Passive smoking | 1.82(1.39, 2.36) | <0.001 | 1.72(1.34, 2.20) | <0.001 | 1.61(1.16, 2.22) | 0.004 | 2.11(1.57, 2.80) | <0.001 |
| Cumulative smoking exposure,  pack-years |  |  |  |  |  |  |  |  |
| 0 | 1 |  | 1 |  | 1 |  | 1 |  |
| 1-19 | 1.29(0.92, 1.80) | 0.14 | 1.03(0.71, 1.45) | 0.9 | 1.31(0.82, 2.05) | 0.2 | 1.48(0.98, 2.18) | 0.053 |
| ≥ 20 | 1.21(0.90, 1.62) | 0.2 | 1.13(0.84, 1.52) | 0.4 | 1.12(0.74, 1.69) | 0.6 | 1.47(1.04, 2.08) | 0.028 |
| Biomass exposure | 1.1(0.75, 1.55) | 0.6 | 0.96(0.66, 1.36) | 0.8 | 0.68(0.38, 1.13) | 0.2 | 1.04(0.68, 1.55) | 0.8 |
| Household cooking | 1.49(1.21, 1.83) | <0.001 | 1.75(1.43, 2.15) | <0.001 | 1.38(1.06, 1.81) | 0.019 | 1.59(1.25, 2.03) | <0.001 |
| Occupational exposure | 1.82(1.42, 2.31) | <0.001 | 1.54(1.20, 1.97) | <0.001 | 1.34(0.95, 1.86) | 0.086 | 1.55(1.16, 2.05) | 0.002 |
| Family history of asthma | 2.21(1.52, 3.13) | <0.001 | 2.45(1.73, 3.39) | <0.001 | 3.15(2.09, 4.60) | <0.001 | 2.94(2.01, 4.18) | <0.001 |
| Season |  |  |  |  |  |  |  |  |
| Winter and summer | 1 |  | 1 |  | 1 |  | 1 |  |
| Spring and autumn | 1.65(1.32, 2.08) | <0.001 | 1.46(1.18, 1.81) | <0.001 | 1.02(0.77, 1.35) | 0.9 | 1.56(1.20, 2.03) | <0.001 |
| Geographic region |  |  |  |  |  |  |  |  |
| North | 1 |  | 1 |  | 1 |  | 1 |  |
| East | 0.57(0.39, 0.81) | 0.002 | 0.72(0.52, 0.98) | 0.04 | 0.39(0.23, 0.62) | <0.001 | 0.63(0.41, 0.93) | 0.026 |
| Northeast | 1.73(1.28, 2.30) | <0.001 | 1.56(1.16, 2.07) | 0.003 | 0.69(0.42, 1.06) | 0.11 | 1.32(0.92, 1.86) | 0.12 |
| Northwest | 0.08(0.03, 0.16) | <0.001 | 0.06(0.02, 0.13) | <0.001 | 0.11(0.04, 0.24) | <0.001 | 0.03(0.01, 0.11) | <0.001 |
| Ambient temperature, ℃ | 1.01(1.0, 1.02) | 0.2 | 1(0.99, 1.02) | 0.8 | 1(0.98, 1.01) | 0.6 | 1.01(0.99, 1.03) | 0.3 |

**Table S2: Estimated risks of each independent variable in the lag0-7 day PM_2.5_ concentration logistic regression model 3.**

|  | Allergic nasal symptoms | | Allergic eye symptoms | | Worsening dyspnea caused  by allergens | | ≥ 2 allergic symptoms | |
| --- | --- | --- | --- | --- | --- | --- | --- | --- |
|  | OR (95%CI) | p-value | OR (95%CI) | p-value | OR (95%CI) | p-value | OR (95%CI) | p-value |
| Lag0-7 day PM_2.5_, per 10 µg/m^3^ | 1.11(1.06, 1.16) | <0.001 | 1.16(1.11, 1.20) | <0.001 | 1.06(1.00, 1.12) | 0.06 | 1.09(1.03, 1.14) | 0.002 |
| Male | 1.28(1.00, 1.63) | 0.053 | 1.1(0.86, 1.39) | 0.4 | 0.83(0.59, 1.14) | 0.3 | 1.01(0.75, 1.35) | >0.9 |
| Age, y | 1.03(1.01, 1.04) | <0.001 | 1.01(1.00, 1.02) | 0.018 | 1.02(1.01, 1.04) | 0.002 | 1.03(1.01, 1.04) | <0.001 |
| BMI group |  |  |  |  |  |  |  |  |
| < 25 kg/m^2^ | 1 |  | 1 |  | 1 |  | 1 |  |
| 25-29.9 kg/m^2^ | 0.83(0.68, 1.00) | 0.051 | 0.91(0.76, 1.09) | 0.3 | 1.16(0.92, 1.47) | 0.2 | 0.9(0.73, 1.12) | 0.4 |
| ≥ 30 kg/m^2^ | 0.93(0.64, 1.32) | 0.7 | 1.22(0.88, 1.65) | 0.2 | 1.24(0.79, 1.88) | 0.3 | 1.1(0.73, 1.60) | 0.6 |
| Education level |  |  |  |  |  |  |  |  |
| Nonschooling or primary school | 1 |  | 1 |  | 1 |  | 1 |  |
| Middle school | 1.57(1.13, 2.22) | 0.009 | 1.81(1.33, 2.49) | <0.001 | 1.29(0.86, 1.98) | 0.2 | 2.16(1.44, 3.35) | <0.001 |
| High school | 2.44(1.74, 3.48) | <0.001 | 2.24(1.63, 3.12) | <0.001 | 2.05(1.36, 3.18) | <0.001 | 3.47(2.29, 5.40) | <0.001 |
| College or higher | 3.47(2.42, 5.07) | <0.001 | 3.14(2.22, 4.49) | <0.001 | 3.16(2.02, 5.04) | <0.001 | 4.45(2.85, 7.13) | <0.001 |
| Passive smoking | 1.82(1.39, 2.36) | <0.001 | 1.76(1.36, 2.25) | <0.001 | 1.61(1.15, 2.21) | 0.004 | 2.11(1.57, 2.80) | <0.001 |
| Cumulative smoking exposure,  pack-years |  |  |  |  |  |  |  |  |
| 0 | 1 |  | 1 |  | 1 |  | 1 |  |
| 1-19 | 1.3(0.92, 1.81) | 0.13 | 1.04(0.73, 1.48) | 0.8 | 1.32(0.82, 2.05) | 0.2 | 1.48(0.99, 2.19) | 0.051 |
| ≥ 20 | 1.23(0.91, 1.64) | 0.2 | 1.17(0.87, 1.56) | 0.3 | 1.13(0.74, 1.70) | 0.6 | 1.49(1.05, 2.10) | 0.023 |
| Biomass exposure | 1.07(0.74, 1.52) | 0.7 | 0.94(0.65, 1.33) | 0.7 | 0.67(0.38, 1.12) | 0.2 | 1.02(0.67, 1.51) | >0.9 |
| Household cooking | 1.48(1.20, 1.83) | <0.001 | 1.75(1.43, 2.15) | <0.001 | 1.38(1.06, 1.81) | 0.017 | 1.59(1.25, 2.03) | <0.001 |
| Occupational exposure | 1.84(1.43, 2.33) | <0.001 | 1.54(1.20, 1.96) | <0.001 | 1.36(0.96, 1.89) | 0.073 | 1.56(1.17, 2.06) | 0.002 |
| Family history of asthma | 2.17(1.49, 3.07) | <0.001 | 2.4(1.70, 3.32) | <0.001 | 3.11(2.06, 4.55) | <0.001 | 2.89(1.98, 4.11) | <0.001 |
| Season |  |  |  |  |  |  |  |  |
| Winter and summer | 1 |  | 1 |  | 1 |  | 1 |  |
| Spring and autumn | 1.73(1.38, 2.17) | <0.001 | 1.54(1.24, 1.91) | <0.001 | 1.05(0.80, 1.39) | 0.7 | 1.62(1.25, 2.10) | <0.001 |
| Geographic region |  |  |  |  |  |  |  |  |
| North | 1 |  | 1 |  | 1 |  | 1 |  |
| East | 0.59(0.40, 0.83) | 0.004 | 0.79(0.57, 1.08) | 0.2 | 0.39(0.23, 0.62) | <0.001 | 0.64(0.42, 0.95) | 0.034 |
| Northeast | 1.77(1.31, 2.36) | <0.001 | 1.7(1.26, 2.27) | <0.001 | 0.69(0.42, 1.06) | 0.11 | 1.34(0.93, 1.90) | 0.1 |
| Northwest | 0.08(0.03, 0.16) | <0.001 | 0.06(0.02, 0.12) | <0.001 | 0.11(0.04, 0.25) | <0.001 | 0.03(0.01, 0.11) | <0.001 |
| Ambient temperature, ℃ | 1.01(1.00, 1.03) | 0.11 | 1.01(1.00, 1.02) | 0.14 | 1(0.98, 1.01) | 0.6 | 1.01(1.0, 1.03) | 0.2 |

**Table S3:** **Estimated risks of each independent variable in the 1-year average PM_2.5_ concentration logistic regression model 4.**

|  | Allergic nasal symptoms | | Allergic eye symptoms | | Worsening dyspnea caused  by allergens | | ≥ 2 allergic symptoms | |
| --- | --- | --- | --- | --- | --- | --- | --- | --- |
|  | OR (95%CI) | p-value | OR (95%CI) | p-value | OR (95%CI) | p-value | OR (95%CI) | p-value |
| PM_2.5_, per 10 µg/m^3^ |  |  |  |  |  |  |  |  |
| 1-year | 1.23(1.14, 1.33) | <0.001 | 1.22(1.14, 1.32) | <0.001 | 1.2(1.09, 1.32) | <0.001 | 1.21(1.11, 1.32) | <0.001 |
| Short-term deviation | 1.07(1.04,1.1) | <0.001 | 1.06(1.03,1.09) | <0.001 | 1.05(1.01,1.09) | 0.014 | 1.06(1.02,1.1) | 0.001 |
| Male | 1.27(0.99, 1.63) | 0.058 | 1.1(0.86, 1.39) | 0.5 | 0.81(0.58, 1.12) | 0.2 | 1(0.74, 1.34) | >0.9 |
| Age, y | 1.03(1.02, 1.04) | <0.001 | 1.02(1.00, 1.03) | 0.006 | 1.03(1.01, 1.04) | <0.001 | 1.03(1.01, 1.04) | <0.001 |
| BMI group |  |  |  |  |  |  |  |  |
| < 25 kg/m^2^ | 1 |  | 1 |  | 1 |  | 1 |  |
| 25-29.9 kg/m^2^ | 0.83(0.68, 1.00) | 0.054 | 0.91(0.76, 1.09) | 0.3 | 1.17(0.92, 1.49) | 0.2 | 0.9(0.73, 1.12) | 0.4 |
| ≥ 30 kg/m^2^ | 0.93(0.64, 1.32) | 0.7 | 1.22(0.88, 1.65) | 0.2 | 1.25(0.79, 1.90) | 0.3 | 1.09(0.73, 1.60) | 0.7 |
| Education level |  |  |  |  |  |  |  |  |
| Nonschooling or primary school | 1 |  | 1 |  | 1 |  | 1 |  |
| Middle school | 1.6(1.15, 2.26) | 0.007 | 1.83(1.35, 2.52) | <0.001 | 1.32(0.88, 2.02) | 0.2 | 2.2(1.46, 3.41) | <0.001 |
| High school | 2.39(1.71, 3.41) | <0.001 | 2.25(1.64, 3.13) | <0.001 | 1.99(1.32, 3.09) | 0.001 | 3.38(2.24, 5.27) | <0.001 |
| College or higher | 3.41(2.37, 4.97) | <0.001 | 3.13(2.22, 4.48) | <0.001 | 3.1(1.98, 4.93) | <0.001 | 4.34(2.78, 6.96) | <0.001 |
| Passive smoking | 1.92(1.46, 2.50) | <0.001 | 1.83(1.42, 2.34) | <0.001 | 1.7(1.21, 2.34) | 0.002 | 2.22(1.65, 2.97) | <0.001 |
| Cumulative smoking exposure,  pack-years |  |  |  |  |  |  |  |  |
| 0 | 1 |  | 1 |  | 1 |  | 1 |  |
| 1-19 | 1.34(0.95, 1.86) | 0.092 | 1.07(0.74, 1.51) | 0.7 | 1.36(0.85, 2.13) | 0.2 | 1.53(1.02, 2.26) | 0.036 |
| ≥ 20 | 1.22(0.91, 1.63) | 0.2 | 1.15(0.85, 1.54) | 0.4 | 1.14(0.75, 1.71) | 0.5 | 1.49(1.05, 2.10) | 0.024 |
| Biomass exposure | 1.05(0.72, 1.49) | 0.8 | 0.92(0.63, 1.30) | 0.6 | 0.65(0.36, 1.09) | 0.12 | 1(0.65, 1.48) | >0.9 |
| Household cooking | 1.43(1.16, 1.77) | <0.001 | 1.68(1.37, 2.07) | <0.001 | 1.32(1.01, 1.73) | 0.044 | 1.53(1.20, 1.96) | <0.001 |
| Occupational exposure | 1.66(1.29, 2.12) | <0.001 | 1.41(1.09, 1.80) | 0.007 | 1.22(0.85, 1.70) | 0.3 | 1.42(1.06, 1.88) | 0.017 |
| Family history of asthma | 2.22(1.53, 3.15) | <0.001 | 2.46(1.74, 3.41) | <0.001 | 3.15(2.09, 4.62) | <0.001 | 2.95(2.02, 4.21) | <0.001 |
| Season |  |  |  |  |  |  |  |  |
| Winter and summer | 1 |  | 1 |  | 1 |  | 1 |  |
| Spring and autumn | 1.61(1.28, 2.02) | <0.001 | 1.42(1.15, 1.77) | 0.001 | 0.98(0.74, 1.30) | 0.9 | 1.51(1.17, 1.97) | 0.002 |
| Geographic region |  |  |  |  |  |  |  |  |
| North | 1 |  | 1 |  | 1 |  | 1 |  |
| East | 0.81(0.53, 1.22) | 0.3 | 1.02(0.70, 1.47) | >0.9 | 0.54(0.30, 0.91) | 0.025 | 0.89(0.55, 1.41) | 0.6 |
| Northeast | 1.96(1.43, 2.66) | <0.001 | 1.78(1.30, 2.39) | <0.001 | 0.78(0.47, 1.22) | 0.3 | 1.5(1.03, 2.16) | 0.031 |
| Northwest | 0.07(0.03, 0.15) | <0.001 | 0.05(0.02, 0.12) | <0.001 | 0.1(0.04, 0.22) | <0.001 | 0.03(0.01, 0.10) | <0.001 |
| Ambient temperature, ℃ | 1(0.99, 1.02) | 0.5 | 1(0.98, 1.01) | 0.6 | 0.99(0.98, 1.01) | 0.3 | 1(0.99, 1.02) | 0.6 |

**Table S4: Estimated risks of each independent variable in the 3-year average PM_2.5_ concentration logistic regression model 4**

|  | Allergic nasal symptoms | | Allergic eye symptoms | | Worsening dyspnea caused  by allergens | | ≥ 2 allergic symptoms | |
| --- | --- | --- | --- | --- | --- | --- | --- | --- |
|  | OR (95%CI) | p-value | OR (95%CI) | p-value | OR (95%CI) | p-value | OR (95%CI) | p-value |
| PM_2.5_, per 10 µg/m^3^ |  |  |  |  |  |  |  |  |
| 3-year | 1.14(1.07, 1.22) | <0.001 | 1.21(1.13, 1.29) | <0.001 | 1.1(1.01, 1.20) | 0.026 | 1.12(1.04, 1.21) | 0.004 |
| Short-term deviation | 1.07(1.04,1.1) | <0.001 | 1.06(1.03,1.09) | <0.001 | 1.05(1.01,1.09) | 0.022 | 1.06(1.02,1.09) | 0.003 |
| Male | 1.28(1.00, 1.64) | 0.048 | 1.11(0.87, 1.41) | 0.4 | 0.83(0.59, 1.14) | 0.3 | 1.01(0.75, 1.36) | >0.9 |
| Age, y | 1.03(1.02, 1.04) | <0.001 | 1.02(1.00, 1.03) | 0.008 | 1.02(1.01, 1.04) | 0.001 | 1.03(1.01, 1.04) | <0.001 |
| BMI group |  |  |  |  |  |  |  |  |
| < 25 kg/m^2^ | 1 |  | 1 |  | 1 |  | 1 |  |
| 25-29.9 kg/m^2^ | 0.82(0.68, 1.0) | 0.046 | 0.9(0.75, 1.08) | 0.3 | 1.16(0.92, 1.47) | 0.2 | 0.9(0.72, 1.11) | 0.3 |
| ≥ 30 kg/m^2^ | 0.92(0.63, 1.30) | 0.6 | 1.2(0.86, 1.63) | 0.3 | 1.23(0.78, 1.86) | 0.3 | 1.08(0.72, 1.58) | 0.7 |
| Education level |  |  |  |  |  |  |  |  |
| Nonschooling or primary school | 1 |  | 1 |  | 1 |  | 1 |  |
| Middle school | 1.58(1.13, 2.23) | 0.008 | 1.81(1.33, 2.49) | <0.001 | 1.29(0.86, 1.99) | 0.2 | 2.17(1.44, 3.36) | <0.001 |
| High school | 2.44(1.74, 3.47) | <0.001 | 2.24(1.63, 3.13) | <0.001 | 2.04(1.35, 3.16) | <0.001 | 3.45(2.29, 5.38) | <0.001 |
| College or higher | 3.47(2.41, 5.05) | <0.001 | 3.13(2.22, 4.47) | <0.001 | 3.13(2.01, 4.99) | <0.001 | 4.42(2.83, 7.08) | <0.001 |
| Passive smoking | 1.88(1.43, 2.45) | <0.001 | 1.86(1.44, 2.38) | <0.001 | 1.65(1.18, 2.28) | 0.003 | 2.17(1.62, 2.90) | <0.001 |
| Cumulative smoking exposure,  pack-years |  |  |  |  |  |  |  |  |
| 0 | 1 |  | 1 |  | 1 |  | 1 |  |
| 1-19 | 1.32(0.94, 1.85) | 0.1 | 1.08(0.75, 1.53) | 0.7 | 1.34(0.83, 2.09) | 0.2 | 1.51(1.01, 2.23) | 0.041 |
| ≥ 20 | 1.23(0.91, 1.64) | 0.2 | 1.17(0.87, 1.57) | 0.3 | 1.13(0.75, 1.70) | 0.5 | 1.49(1.05, 2.10) | 0.023 |
| Biomass exposure | 1.07(0.74, 1.52) | 0.7 | 0.93(0.64, 1.31) | 0.7 | 0.67(0.37, 1.12) | 0.15 | 1.02(0.67, 1.52) | >0.9 |
| Household cooking | 1.48(1.21, 1.83) | <0.001 | 1.74(1.42, 2.14) | <0.001 | 1.38(1.06, 1.80) | 0.019 | 1.59(1.25, 2.03) | <0.001 |
| Occupational exposure | 1.77(1.37, 2.25) | <0.001 | 1.44(1.12, 1.84) | 0.004 | 1.3(0.92, 1.82) | 0.13 | 1.51(1.13, 1.99) | 0.005 |
| Family history of asthma | 2.23(1.53, 3.15) | <0.001 | 2.49(1.76, 3.45) | <0.001 | 3.16(2.09, 4.63) | <0.001 | 2.95(2.02, 4.21) | <0.001 |
| Season |  |  |  |  |  |  |  |  |
| Winter and summer | 1 |  | 1 |  | 1 |  | 1 |  |
| Spring and autumn | 1.63(1.30, 2.05) | <0.001 | 1.41(1.14, 1.75) | 0.002 | 1(0.76, 1.33) | >0.9 | 1.54(1.19, 2.00) | 0.001 |
| Geographic region |  |  |  |  |  |  |  |  |
| North | 1 |  | 1 |  | 1 |  | 1 |  |
| East | 0.67(0.44, 0.99) | 0.051 | 0.98(0.68, 1.39) | >0.9 | 0.43(0.25, 0.73) | 0.002 | 0.73(0.45, 1.13) | 0.2 |
| Northeast | 2(1.42, 2.80) | <0.001 | 2.11(1.50, 2.93) | <0.001 | 0.76(0.45, 1.24) | 0.3 | 1.51(1.00, 2.24) | 0.047 |
| Northwest | 0.07(0.03, 0.15) | <0.001 | 0.05(0.02, 0.12) | <0.001 | 0.1(0.04, 0.23) | <0.001 | 0.03(0.01, 0.10) | <0.001 |
| Ambient temperature, ℃ | 1.01(0.99, 1.02) | 0.4 | 1(0.98, 1.01) | 0.7 | 0.99(0.98, 1.01) | 0.4 | 1.01(0.99, 1.02) | 0.5 |

**Table S5: Estimated risks of each independent variable in the 5-year average PM_2.5_ concentration logistic regression model 4**

|  | Allergic nasal symptoms | | Allergic eye symptoms | | Worsening dyspnea caused  by allergens | | ≥ 2 allergic symptoms | |
| --- | --- | --- | --- | --- | --- | --- | --- | --- |
|  | OR (95%CI) | p-value | OR (95%CI) | p-value | OR (95%CI) | p-value | OR (95%CI) | p-value |
| PM_2.5_, per 10 µg/m^3^ |  |  |  |  |  |  |  |  |
| 5-year | 1.13(1.06, 1.20) | <0.001 | 1.19(1.12, 1.26) | <0.001 | 1.09(1.00, 1.18) | 0.041 | 1.11(1.03, 1.19) | 0.007 |
| Short-term deviation | 1.07(1.04,1.1) | <0.001 | 1.06(1.03,1.09) | <0.001 | 1.05(1.01,1.09) | 0.021 | 1.06(1.02,1.09) | 0.003 |
| Male | 1.28(1.00, 1.64) | 0.047 | 1.11(0.87, 1.41) | 0.4 | 0.83(0.59, 1.14) | 0.3 | 1.01(0.75, 1.36) | >0.9 |
| Age, y | 1.03(1.02, 1.04) | <0.001 | 1.02(1.00, 1.03) | 0.008 | 1.02(1.01, 1.04) | 0.001 | 1.03(1.01, 1.04) | <0.001 |
| BMI group |  |  |  |  |  |  |  |  |
| < 25 kg/m^2^ | 1 |  | 1 |  | 1 |  | 1 |  |
| 25-29.9 kg/m^2^ | 0.82(0.68, 0.99) | 0.043 | 0.9(0.75, 1.08) | 0.3 | 1.16(0.91, 1.47) | 0.2 | 0.9(0.72, 1.11) | 0.3 |
| ≥ 30 kg/m^2^ | 0.92(0.63, 1.30) | 0.7 | 1.2(0.87, 1.63) | 0.3 | 1.24(0.78, 1.87) | 0.3 | 1.08(0.72, 1.58) | 0.7 |
| Education level |  |  |  |  |  |  |  |  |
| Nonschooling or primary school | 1 |  | 1 |  | 1 |  | 1 |  |
| Middle school | 1.58(1.14, 2.24) | 0.008 | 1.82(1.34, 2.51) | <0.001 | 1.3(0.87, 1.99) | 0.2 | 2.18(1.45, 3.37) | <0.001 |
| High school | 2.45(1.75, 3.48) | <0.001 | 2.25(1.64, 3.13) | <0.001 | 2.04(1.35, 3.17) | <0.001 | 3.46(2.29, 5.39) | <0.001 |
| College or higher | 3.49(2.43, 5.08) | <0.001 | 3.15(2.23, 4.50) | <0.001 | 3.14(2.01, 5.01) | <0.001 | 4.44(2.84, 7.12) | <0.001 |
| Passive smoking | 1.86(1.41, 2.42) | <0.001 | 1.83(1.42, 2.35) | <0.001 | 1.64(1.17, 2.26) | 0.003 | 2.15(1.60, 2.87) | <0.001 |
| Cumulative smoking exposure,  pack-years |  |  |  |  |  |  |  |  |
| 0 | 1 |  | 1 |  | 1 |  | 1 |  |
| 1-19 | 1.32(0.93, 1.84) | 0.11 | 1.08(0.75, 1.53) | 0.7 | 1.33(0.83, 2.08) | 0.2 | 1.51(1.00, 2.23) | 0.043 |
| ≥ 20 | 1.22(0.91, 1.63) | 0.2 | 1.17(0.87, 1.56) | 0.3 | 1.13(0.74, 1.70) | 0.6 | 1.49(1.05, 2.10) | 0.024 |
| Biomass exposure | 1.08(0.75, 1.53) | 0.7 | 0.94(0.65, 1.33) | 0.7 | 0.67(0.37, 1.12) | 0.2 | 1.03(0.67, 1.53) | 0.9 |
| Household cooking | 1.47(1.20, 1.82) | <0.001 | 1.72(1.40, 2.11) | <0.001 | 1.37(1.05, 1.79) | 0.022 | 1.58(1.24, 2.02) | <0.001 |
| Occupational exposure | 1.78(1.39, 2.27) | <0.001 | 1.46(1.13, 1.86) | 0.003 | 1.32(0.93, 1.83) | 0.11 | 1.52(1.14, 2.01) | 0.004 |
| Family history of asthma | 2.23(1.54, 3.15) | <0.001 | 2.49(1.76, 3.44) | <0.001 | 3.16(2.09, 4.62) | <0.001 | 2.95(2.02, 4.21) | <0.001 |
| Season |  |  |  |  |  |  |  |  |
| Winter and summer | 1 |  | 1 |  | 1 |  | 1 |  |
| Spring and autumn | 1.62(1.29, 2.04) | <0.001 | 1.4(1.13, 1.73) | 0.002 | 1(0.76, 1.33) | >0.9 | 1.53(1.18, 1.99) | 0.001 |
| Geographic region |  |  |  |  |  |  |  |  |
| North | 1 |  | 1 |  | 1 |  | 1 |  |
| East | 0.63(0.42, 0.93) | 0.022 | 0.92(0.64, 1.29) | 0.6 | 0.42(0.24, 0.69) | 0.001 | 0.69(0.43, 1.06) | 0.1 |
| Northeast | 1.91(1.35, 2.67) | <0.001 | 2.03(1.45, 2.82) | <0.001 | 0.74(0.44, 1.20) | 0.2 | 1.45(0.96, 2.16) | 0.071 |
| Northwest | 0.08(0.03, 0.16) | <0.001 | 0.06(0.02, 0.13) | <0.001 | 0.11(0.04, 0.24) | <0.001 | 0.03(0.01, 0.11) | <0.001 |
| Ambient temperature, ℃ | 1.01(0.99, 1.02) | 0.4 | 1(0.98, 1.01) | 0.6 | 0.99(0.98, 1.01) | 0.4 | 1.01(0.99, 1.02) | 0.5 |
